# Self-Healing Materials-Based Electronic Skin: Mechanism, Development and Applications

**DOI:** 10.3390/gels8060356

**Published:** 2022-06-06

**Authors:** Jingjie Chen, Lei Wang, Xiangou Xu, Guming Liu, Haoyan Liu, Yuxuan Qiao, Jialin Chen, Siwei Cao, Quanbin Cha, Tengjiao Wang

**Affiliations:** 1 Frontiers Science Center for Flexible Electronics (FSCFE), Xi’an Institute of Flexible Electronics (IFE) & Xi’an Institute of Biomedical Materials and Engineering (IBME), Northwestern Polytechnical University (NPU), Xi’an 710072, China; chenjingjie@mail.nwpu.edu.cn (J.C.); iamlwang@mail.nwpu.edu.cn (L.W.); xiangou@mail.nwpu.edu.cn (X.X.); 2019303771@mail.nwpu.edu.cn (G.L.); iamyxqiao@mail.nwpu.edu.cn (Y.Q.); 2 Key Laboratory of Flexible Electronics of Zhejiang Province, Ningbo Institute of Northwestern Polytechnical University, Ningbo 315103, China; 3 Queen Mary University of London Engineering School, Northwestern Polytechnical University (NPU), Xi’an 710072, China; 771484614@mail.nwpu.edu.cn (J.C.); caosiwei@mail.nwpu.edu.cn (S.C.); 19939815433@mail.nwpu.edu.cn (Q.C.); 4 Department of Computer Science and Computer Engineering, University of Arkansas, Fayetteville, AR 72701, USA; hl002@uark.edu; 5 Honors College, Northwestern Polytechnical University (NPU), Xi’an 710072, China

**Keywords:** electronic skin, hydrogels, self-healing, wearable sensors, flexible electronics

## Abstract

Electronic skin (e-skin) has brought us great convenience and revolutionized our way of life. However, due to physical or chemical aging and damage, they will inevitably be degraded gradually with practical operation. The emergence of self-healing materials enables e-skins to achieve repairment of cracks and restoration of mechanical function by themselves, meeting the requirements of the era for building durable and self-healing electronic devices. This work reviews the current development of self-healing e-skins with various application scenarios, including motion sensor, human–machine interaction and soft robots. The new application fields and present challenges are discussed; meanwhile, thinkable strategies and prospects of future potential applications are conferenced.

## 1. Introduction

Electronic skin (e-skin) is a highly integrated and ingenious electronic system that can convert various external stimuli such as pressure, deformation and humidity into electronic signals. It also can imitate some basic functions of human skin including the capability of stretching, self-healing and versatile senses [1,2,3]. It has shown large potential for application in wearable healthcare sensors, tactile devices, robotic artificial skin, prostheses and implantable medical devices [4,5]. To date, e-skin has achieved flexibility, low weight, miniaturization and multifunctionality [6,7]. However, under the action of many factors such as stretching, twisting, cutting, compression and excessive usage, the abrasion, degradation or mechanical damage of e-skins is caused inevitably. This weakens its performance and results in failure, followed by seriously reduced reliability and shortened service life [8,9,10,11]. The high integration of e-skin makes it difficult and costly to maintain after damage. In addition, the wide application of e-skin will generate a large amount of electronic waste, causing environmental pollution [12]. Therefore, designing e-skin with a self-healing ability is an ideal way to address these problems.

In nature, after being subjected to a certain range of external mechanical damage, organisms can repair themselves and restore their original structures and functions [13,14]. Inspired by this, researchers have conducted many studies on artificial self-healing systems [15,16]. Self-healing e-skin combines self-healing materials with electronic devices and has become one of the main research areas of artificial self-healing systems. To date, scientists have proposed a new requirement for the ideal e-skin: that it can restore not only structural and mechanical properties, but even its electrical properties and functions to improve the durability, reliability and safety of the e-skin [17,18].

Current self-healing e-skin application scenarios involve soft robotics systems, health monitoring devices, artificial intelligence and communication devices. Focused on the self-healing property of these e-skins, we summarize its recent research developments here, with comprehensive consideration from commonly used self-healing materials and self-healing mechanisms to device design principles and resultant performances. Additionally, future perspectives and possible strategies for existing challenges are emphasized, which can inspire the development of high-performance and multifunctional self-healing e-skins.

## 2. Self-Healing Mechanisms

At present, the healing mechanisms of self-healing materials include autonomous and nonautonomous systems. Non-autonomous self-healing materials can be incorporated into the material system and usually require external stimulation, such as light, heat or pH, to achieve the healing effect. However, autonomous self-healing materials can initiate the self-healing process without any external stimuli or triggers [19]. On the whole, self-healing materials can be categorized into intrinsic and extrinsic systems according to the self-healing principles.

### 2.1. Extrinsic Self-Healing Materials

Materials with the extrinsic self-healing nature usually repair the damage with pre-added healing agents, which typically contain catalysts and reactive precursors within self-healing materials [19]. Healing agents are usually stored in the microcapsule or microvascular network based on polymer matrixes. Upon damage, the containers will rupture while the healing agents are delivered to the crack to achieve the repairing effect by polymerization or chemical reactions. In addition, since the trigger that activates healing is the destructive force that causes the encapsulations to break and the healing agents to be released, some external stimuli such as heat or light are usually required to promote the self-healing behavior.

#### 2.1.1. Microcapsule Embedment

The capsules’ inserted compounds with reactive groups or healing functionality could carry out chemical reactions leading to materials healing, which include various processes such as ring-opening metathesis polymerization, crosslinking reactions, cyclo-addition, cyclo-reversion or mechanochemical catalytic activation [19]. The healing agent will flow by capillary action and reach the fracture location when the microcapsules fracture and break, where the healing agent diffuses in the two fracture surfaces by means of surface tension to achieve the purpose of healing [20]. Furthermore, the precursor interacts with the adjacent intercalated catalyst to form a network that prevents further crack growth and restores mechanical integrity by continuing the above reaction.

White et al. reported the first structural polymer material whose microencapsulated healing agent has the ability to heal damage autonomously [21]. Cracks in the matrix caused the microcapsules to rupture and release a dicyclopentadiene (DCPD) monomer, then the healing agent diffused to the crack interface under the capillary action. Thereafter, the DCPD monomer is contacted with the embedded catalyst to initiate ring-opening polymerization, thereby repairing the crack. Structural design, preparation methods and self-healing mechanisms are the key factors of the research on microencapsulation self-healing systems. Among them, the preparation technologies of microcapsules, including in situ/interfacial polymerization, melt dispersion, sol–gel reaction, microemulsion polymerization and the acid wash emulsion template method, have matured [13,22]. Furthermore, the wide selection of self-healing agents, including monomeric, catalytic and liquid metal alloys, enables self-healing composites with many specific properties. Blaiszik et al. designed nanocapsules with an average size of 220 nm, and Kirkby et al. further modified this design [23,24]. The incorporated shape memory alloy (SMA) filaments were contained in the composites to reach the reduction of crack volume and improvement of crack-filling factor. However, a repeated healing process was only possible after the first damage if the healing agents remained in the damaged region [25].

#### 2.1.2. Microvascular Embedment

To address the limitation that self-healing materials can only heal once, researchers designed a self-healing microvascular system. Inspired by the respiratory system of living organisms, microvascular technology was utilized. Composite materials for self-healing properties are usually composed of very fine hollow fibers and a mesoporous structural compound, which can effectively increase the lifespan of the incorporated structural material and achieve multiple self-healing processes [26]. When damage occurs, the interconnected complex network of hollow vessels or canals is retained due to the embedment of microvascular technologies, and the healing agent is incorporated. This state continues until the fractures are repaired [27,28].

Cuvellier et al. have been researching tailored pullulan nanofibers by electrospinning to replace the microcapsules and then improve the mechanical properties and self-healing abilities of e-skins [29]. The selection of healing agents will affect the performance of the healing process. Moreover, this group has studied four types of healing systems to improve self-healing and found that a higher glass transition temperature leads to a higher healing capability [30]. However, the consumption of the healing agent is irreversible, directly making it impossible for a microencapsulated self-healing system to achieve multiple healings at the same site. In order to improve the self-healing capacity of a vascular system, vascular network systems with different dimensions are designed, such as one-dimensional (1D), two-dimensional (2D) or three-dimensional (3D) networks [31].

White et al. developed a biomimetic self-healing microvascular network [32,33]. Mimicking biological vasculature, they constructed a 3D hollow microtubule network in the matrix filled with healing agents. The self-healing mechanism of the microvascular network was similar to that of microcapsules, but the most important difference was that the microvascular network structure could store more self-healing agents and realize multiple self-healings. After that, White et al. further developed a microcapsule–microvascular self-healing composite system for repairing multiscale damage caused by impact puncture [34,35,36]. Although the authors achieved multiple self-healing goals, the fabrication process of such a self-healing system was very complex and time-consuming, resulting in a high cost.

### 2.2. Intrinsic Self-Healing Materials

Unlike the extrinsic self-healing mode, the intrinsic self-healing mode does not require a self-healing agent and can achieve multiple reversible self-healings. Compared with the extrinsic self-healing mode, it has a more stable and reliable self-healing ability and avoids complex encapsulation and dispersion steps [18]. Overall, the intrinsic self-healing mode depends on the recombination of internal reversible dynamic covalent bonds or the reconstruction of non-covalent bonds between cracked interfaces [37]. Reversible dynamic covalent bonds, such as imine bonds [38], disulfide bonds [39], acylhydrazone bonds [40], carbon–carbon double bonds [41], urea bonds [42] and so on, possess stronger bond energy than noncovalent bonds, which makes it possible for self-healing materials to realize outstanding mechanical properties and stable self-healing abilities. To develop new materials with dynamic crosslinking properties, reversible covalent bonds are commonly used, including the Diels–Alder (DA) reaction, disulfide exchange reaction and transesterification reaction. However, most intrinsic self-healing materials with covalent bonds usually require external stimuli to generate the healing process due to the slow formation of covalent bonds, such as heat, light and pH changes.

The forces in supramolecular chemistry are reversible dynamic non-covalent bonds dominated by hydrogen bonds [43], π–π stacking [44], hydrophobic interactions [45], host–guest interactions [46] and metal–ligand bonds [47], which endow materials with self-healing functions mainly through inter- and/or intramolecular interactions between the specific functional moieties of the polymer chains. These forces are relatively weak compared to covalent bonds but have strong advantages in forming dynamic systems. Different from networks formed by covalent bonds, those formed by non-covalent bonds can be reversibly reconfigured from fluid-like, low-density and high-free-volume states to solid-like, low-free-volume, elastic and plastic networks.

#### 2.2.1. Hydrogen Bonds

Crosslink elastomeric networks with self-healing capacities commonly contain hydrogen bonds [48,49]. The hydrogen bond energy is theoretically about 10 kJ mol^−1^; the specific value depends on the electron donor and acceptor. Weaker bond energies allow hydrogen bonds to reform with less energy after being broken. Low-bond-energy crosslinking negatively affects the mechanical strength, creep properties and strain recovery of the material, but its orientation and higher crosslinking concentration per unit volume provide acceptable properties for the material [50]. Multiple weak interactions between hydrogen bond units form a supramolecular polymer network that can greatly improve the mechanical strength and structural stability of the material, while the network can prompt the reform of the hydrogen bonds [51]. After the damage occurs in the material, the hydrogen bonds will resist external force and break. When the cross-sections of the material come into contact again, the molecules drift dynamically and the hydrogen bonds are reformed. At that point, the properties of the material return to their original state. The material based on hydrogen-bond self-healing allows the material to undergo multiple cycles of damage and healing [52]. Because of these advantages, the study of materials utilizing hydrogen bonding for self-healing has received extensive attention.

Inspired by multiple hydrogen bonds linked by the double helix structure of deoxyribonucleic acid (DNA), to obtain an ultrafast self-healing ability and autonomy, Cao et al. used biologically derived carboxyl cellulose nanocrystals (C-CNC) with shell chitosan (CT)-decorated epoxy natural rubber (ENR) latex to construct multiple hydrogen bond interactions [53,54]. The synthesized sample multiple-hydrogen-bonding elastomer (MHBE) showed an ability to self-heal in real time in just 15 s, which is much faster than most self-healing elastomers reported previously [54]. Moreover, it still showed high toughness and high recovery efficiency after three fractures (*η* ≈ 93%). In the bending failure re-healing experiment of the sample, the resistance of the self-healing sample increased only slightly (not more than one order of magnitude) after bending more than 20,000 times. SEM images exhibited that the damaged interface was completely healed without scarring, confirming the complete self-healing process.

#### 2.2.2. Thermo-Reversible Covalent Bonds

Materials accomplished intrinsic self-healing by thermo-reversible covalent bonds through the DA reaction mostly [55,56,57]. The reaction could enable the crosslinks between the diene and dienophile groups, rendering a firm network [57]. When the crack occurs, the healing mechanism would be initiated by the increase in the temperature, which induces the reaction in the direction of exotherm and breaks the crosslinks. As a result, the reactive diene and dienophile groups would gather, increasing the concertation. Once the reactants are facilitated to contact closely and cool at a lower temperature, the reaction equilibrium would move towards the opposite direction to establish the network caused by the formation of crosslinks [57]. The mechanism of introducing the structure characterized by thermo-reversible covalent bonds has been proven to be a potential solution for efficient self-healing. Terryn and his team have applied this mechanism in the realm of the construction of soft robotics such as soft grippers, soft hands and artificial muscles [58]. The result showed that the self-healing efficiencies of the materials reached up to 98% with no weak spots.

#### 2.2.3. Photo-Reversible Bonds

Photo-reversible self-healing bonds are achieved by the introduction of photo-reversible bond features with the ability to repair the damage locally, which makes them superior in this realm [48]. In the photo-reversible reaction, the olefinic compounds decompose into cyclobutane-type compounds by dimerization reactions with irradiation of UV light above 300 nm wavelength. Interestingly, this kind of conversion can be inverted with the exposure of UV light with a shorter wavelength [59]. Recently, the introduction of photo-reversible bonds has been applied to the research of e-skins effectively. George P. Simon and Kei Saito selected four photo-reversible crosslinking epoxies by curing a series of commercially available epoxies by using two anthracene-based diamine crosslinking agents [60]. The results show that the repair effect is due to the photo-reversible cracking of anthracene dimers in the center of the crosslinking agents, which leads to the transition from the rigid and flexible phase to the mobile phase and then restores to the rigid and flexible phase after filling the damaged part. The research confirmed the existence of this mechanism in polymer networks through analytical tests and explained in detail the healing effects of photo-reversible reactions.

#### 2.2.4. Exchange Reaction Covalent Bond

Exchange covalent bond reactions can lead to intrinsic healing. Once the covalent bonds break with the external stimulus, the new covalent bond of a similar type forms simultaneously to heal the damage effectively [61]. During the exchange reaction, the number of bonds keeps a constant. However, the rate of the exchanging process is accelerated with the increase in temperature. While the e-skin is damaged, at the location of the fracture, the exchange reaction introduces a new covalent bond which compensates for the breakage of the original bond [62,63]. Chen Y. and Tang H. propose a facile approach to preparing permanently crosslinked yet self-healing and recyclable diene rubber by programming dynamic boronic ester linkages into the network, which is synthesized through a one-pot thermally initiated thiol-ene “click” reaction between a novel dithiol-containing boronic ester crosslinker and commonly used styrene–butadiene rubber without modifying the macromolecular structure [64]. The samples they prepared were covalently crosslinked, and their mechanical strengths can be simply adjusted by varying the content of the boronic ester. Owing to the transesterification of boronic ester bonds, the samples can alter network topologies, endowing the materials with self-healing abilities and malleability.

#### 2.2.5. Ionic (Coordination) Complexes

Ionic interactions were also able to take part in the self-healing polymers based on reversible crosslinked networks [65,66]. Comparable with hydrogen bonds because of their saturability and direction abilities leading to the loss of self-healing abilities under room temperature, ionic bonds are a strong electrostatic interaction between the opposite charges’ atoms or groups [67]. The ionic bond is not only unsaturated but also directional, so the ionomers are formed due to the charges being arranged with as many as possible around an opposite charge. Self-healing is achieved by contacting opposite charges on the fracture surface and then through the attraction of the ionic interaction of opposite charges.

To achieve the self-healing ability of rubbers, one of the most effective methods is introducing a reversible ionic network. The substance named “lutiods” is contained in natural rubber that could form protein dimers under the influence of Ca^2+^ ions. Inspired by the rubber tree itself, Nuur Laila Najwa Thajudin introduced Zn^2+^ to substitute Ca^2+^, which enabled the formation of a reversible network and endowed the natural rubber with the self-healing ability [68]. This method provided a new direction for forming self-healing natural rubber.

#### 2.2.6. π–π Stacking Interactions

The π–π stacking interactions between π orbitals of aromatic rings are often regarded as an extension of coordination chemistry [69]. These π–π stacking interactions, which are highly dependent on chemical structure and stereochemistry, are essentially a kind of reversible non-covalent bond interaction as well as a vital factor for the self-healing properties of materials [70]. For example, dynamic reversible π–π stacking interactions of the fluorenyl rings enabled the Fmoc-grafted chitosan and Fmoc peptide (FC/FI) hybrid hydrogel to exhibit excellent injectable and self-healing properties, which can be used to repair spinal cord injuries [71]. The combination of π–π stacking interactions, metal-coordination chemistry and/or H-bonding has been used for the design of several self-healing elastomers. For instance, the π–π stacking interactions between Pt–Pt and a cyclometalated platinum (II) complex was able to form a high stretchable and self-healable polydimethylsiloxane (PDMS) backbone [72]. Compositing with metal nanoparticles also has value to be developed. For example, π–π stacking interactions between pyrene-functionalized gold nanoparticles and the polymer matrix, including a blend of pyrene-functionalized poly amide (π–electron donor) and polydiimide (π–electron acceptor), can result in self-healing [73]. In addition, thermally triggered self-healing can be achieved under the conjunction of π–π stacking interactions and H-bonding [74]. 

In addition, the combination of π–π stacking interactions and H-bonding can be a feasible way to design UV-triggered self-healing materials. For example, polydopamine (PDA)-containing benzene rings and polar groups, including hydroxyl, amino and carbonyl groups, can enhance the compatibility of SiO_2_/PDA hybrid microcapsules and maintain a satisfactory self-healing ability due to π–π stacking interactions between benzene rings and H-bonding between these polar groups [75]. Conductive self-healing polymers such as lithium–sulfur battery anodes can be considered integrating ionic moieties into π–π stacking systems, which may provide technical opportunities for development [76]. The unique multi-crosslinked double-network structure including Schiff-base dynamic covalent bonding, hydrogen bonding and π–π stacking interactions endows the hydrogel with both improved injection abilities and mechanical performance while self-healing faster than single-network hydrogels [77]. Hydrogels assembled via π–π stacking interactions, hydrogen bonds, dynamic borate ester bonds and cation coordination possess tunable mechanical properties, excellent self-healing properties and reversible degradation behavior in response to pH, glucose and ion concentration [78]. However, the bond energy (8–12 kJ/mol) of π–π stacking is lower than the hydrogen bond energy and the crosslinks formed are relatively weak, which makes it difficult to guarantee the strengths and elastic recovery of materials [79]. Therefore, the application of π–π stacking interactions in the field of self-healing flexible electronics is also limited. 

#### 2.2.7. Metal–Ligand Interactions

By acting as reversible physical crosslinks, metal–ligand (coordination) complexes can also achieve supramolecular network formation [80,81]. Similar to the clusters in ionomers, metal–ligand coordination complexes are formed between metal ions and appropriate ligands, linking the polymer chains together [82]. Since the charge on the metal ion is usually much bigger than that of the ligand molecule, the ionic interaction is correspondingly stronger than the dipole–ion interaction, that is, the metal–ligand crosslinking complexes are much weaker. This endows them with the ability to heal macroscopic cracks at ordinary temperatures without the need for an external stimulus. Another appealing feature associated with metal–ligand interactions is that the association strengths will different when coordinating with different kinds of metal ions and ligand substitutes.

#### 2.2.8. Host–Guest Interactions

Guest–host chemistry is commonly used in constructing self-healing polymers. For instance, the hydrophobic cavity of β-cyclodextrin can accommodate a diverse range of guest moieties [83,84]. When a surface containing one cyclodextrin host meets the other guest molecules, host–guest interactions will occur and result in bonding [85]. The supramolecular polymers possess multiple molecular recognition sites, which are realized by various water-soluble polymer backbones modified by β-cyclodextrin hosts and hydrophobic adamantine as side-chain guests. This facile approach yields a transparent, flexible and tough hydrogel, which can self-heal regardless of wet or dry states [86]. Supramolecular hydrogels prepared from modified hyaluronic acid and adamantine or β-cyclodextrin are capable of forming intermolecular host–guest bonds rapidly [87]. Changing the concentration and ratio of host and guest components can adjust the mechanical properties of the system [88].

#### 2.2.9. High-Temperature Transition Phase

The high-temperature transition phase could be used for the self-healing of thermoplastic elastomers with high physical crosslinks. This is because heat treatment could enhance molecular mobility and assist the reformation of dynamic bonds so that the high-temperature transition phase is a possible method for the healing of harder polymers [89]. The self-healing ability is formed by a fully reversible process of fracture upon heating and then reconnection upon cooling. This process does not require additional ingredients such as a catalyst, other conditions, or a special surface treatment [90].

Naoko Yoshiea developed a method that could recover mechanical properties of self-healing polymers by mild heating [89]. Through the reversible DA reaction of poly (2,5-furandimethylene succinate-co-propylene succinate) and bismaleimide, several kinds of bio-based network polymers were formed. Additionally, the glass transition temperature, Tg, could be controlled by changing the amount of bismaleimide added to the copolymer. With the experiment of self-healing, there is a clear relationship between healing ability and glass transition temperature, and, with temperatures above Tg = 15 °C, the method would have a good balance between mild healing conditions and recovery of high mechanical strength.

## 3. Self-Healing Electronic Skin

Fully autonomous self-healing polymers will not require human intervention; the cost will become lower. The current self-healing methods have greatly improved upon the traditional weak kinetic bond, which makes it possible to use these polymers as the basis of e-skin in large-scale applications in motion sensors, soft robots, human–computer interactions and other fields.

### 3.1. Motion Sensors

**Figure 1:**
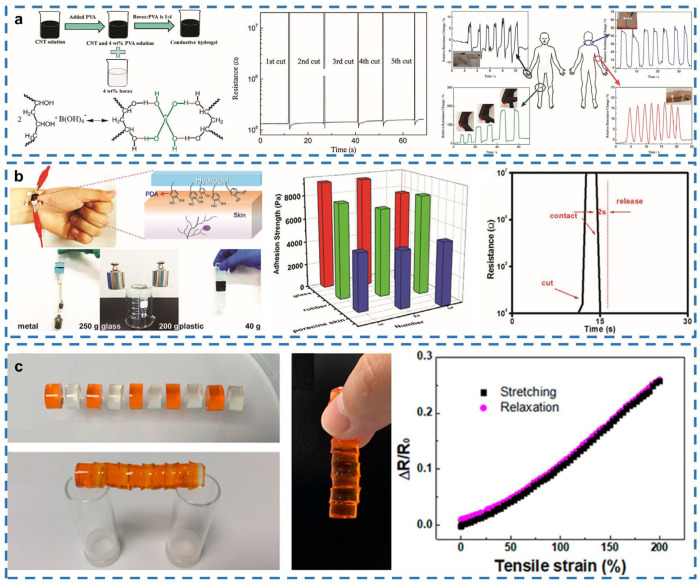
Self-healing motion sensors. (**a**) Conductive SWCNT–hydrogel based self-healing strain sensor. Reprinted with permission from [98]. Copyright 2017, WILEY-VCH. (**b**) Mussel-inspired conductive-hydrogel-based self-healing epidermal sensor. Reprinted with permission from [99]. Copyright 2017, WILEY-VCH. (**c**) Gellan gum hybrid-hydrogel-based self-healing strain sensor. Reproduced with permission from [91]. Copyright 2020, American Chemical Society.

Motion sensors are one of the potential applications of conductive and healable e-skin [91]. When e-skin is compressed or stretched, its resistance will change proportionally along with the deformation of the e-skin, exhibiting fluctuations in the current. In this way, human motion can be detected and transferred into electrical signals. Since self-healing ability and high stretchability are highly desired by wearable devices, stretchable, self-healing and conductive hydrogels have attracted considerable attention. Currently, the representative strain sensors as graphene or semiconductor/metal can only be stretched limitedly, not exceeding strain of 200%, and the healing ability of them is also poor [92,93,94,95,96,97]. Since human motion is usually complex and subtle, it is necessary for wearable strain sensors to have a considerable stretchability with fast response. Additionally, to expand the lifetime of the strain sensor and to adapt to a variety of different environments, structural material with a good self-healing ability will be preferred. Cai et al. have researched SWCNT/hydrogels and provided a possibility to develop further applications as strain sensors [98]. Based on the existence of hydrogen bonds, the porous structure of the SWCNT and polymeric network inside the SWCNT/hydrogel, this hydrogel has indicated a swift electrical healing speed (within 3.2 s) and a high self-healing efficiency (98%). Moreover, it is capable of bearing the strain of 1000% or the strain of 700% 1000 times. The resistance keeps constant even with 5 cutting–healing cycles, which means it has good healing stability and reliability in human-motion-monitoring applications (Figure 1a).

However, in order to have an accurate detection of both large-scale and subtle human activities, high accuracy of strain sensors is needed. Liao et al. have used carbon nanotubes, supramolecular, biocompatible polyvinyl alcohol and polydopamine to synthesize a conductive and healable PVA–FSWCNT–PDA hydrogel [99]. Due to the catechol groups on the PDA chain, this hydrogel has special adhesive properties and can easily adhere to human skin. The experiments have shown the cracked hydrogel can be recovered to 99% of its original state within 2 s; meanwhile, the electrical resistance keeps at the same level and the wearable device made by this healable hydrogel still exhibits high sensitivity to subtle motion after the healing process (Figure 1b).

Though the high sensitivity and high conductivity are accomplished by this hydrogel, its stretchability is limited. Liu et al. have combined a physically crosslinked network with a chemically crosslinked one to strengthen the mechanical properties of the gellan gum hybrid hydrogel [91]. Hydrogen bonds and ionic association exist in the gellan gum network; preferred mechanical properties have been achieved in this hybrid hydrogel, such as strain at break of 1700% and fracture energy of 7840 J/m^2^. Due to the dual network, even in the presence of a notch, the hydrogel can stand the strain of 1000% while the notch keeps stable. Additionally, the native thermo-responsive property allows the gellan gum gel network to be reformed through the heating–cooling process to achieve better properties. With the thermo-reversible gelation of gellan gum and Na^+^, K^+^ and Ca^2+^ ions in gellan gum powder, the self-healing ability and conductivity can be ensured. The experiment results of this hybrid hydrogel have proved that the curves of resistance vs. time almost overlap after 50 cycles of stretching and releasing, indicating good durability of the fabricated strain sensor (Figure 1c).

### 3.2. Human–Machine Interaction

Human–machine interaction (HMI) as a method to realize the communication of humans and machines could make work efficient and transform the human lifestyle [100,101]. Compared to traditional HMI devices that require large numbers of letter buttons and mice [102], new HMI devices are much more intelligent, adopting human posture or actions as input sources. However, a huge challenge lies in how to fit the irregular surface of human skin. The crosslinked 3D network structure and rich water content of e-skin could make sure it is attached to any surface, such as human skin. Hence, the self-healing ability of the e-skin is highly desired for the future development of HMI systems.

**Figure 2:**
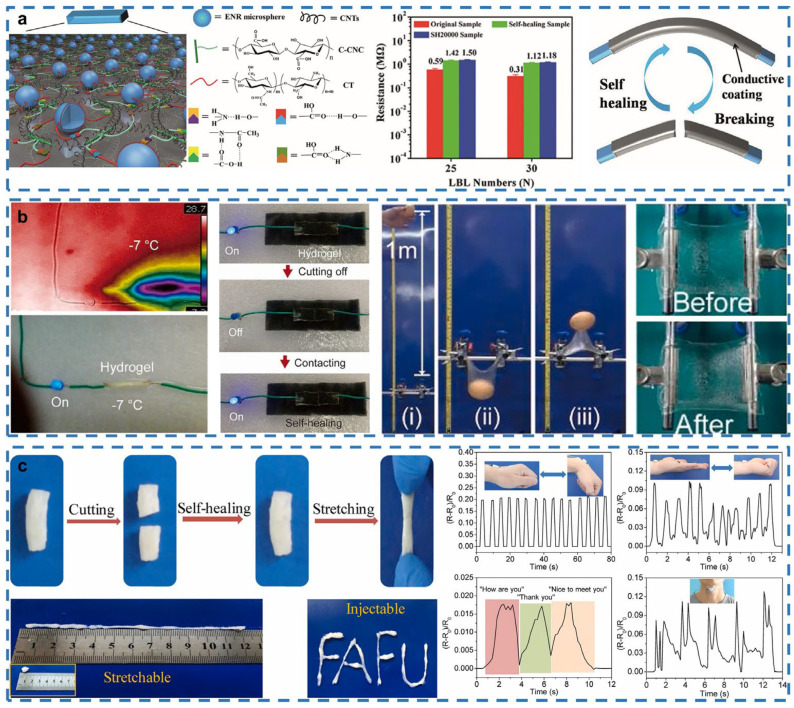
Self-healing human–machine interaction. (**a**) Multiple-hydrogen-bonding-elastomer-based self-healing sensor. Reprinted with permission from [55]. Copyright 2017, WILEY-VCH. (**b**) Conductive cold-resistant and elastic hydrogel-based bionic skin. Reprinted with permission from [104]. Copyright 2021, Elsevier. (**c**) Conductive poly(N-vinylpyrrolidone)/gallic acid hydrogel-based wearable sensor. Reproduced with permission from [106]. Copyright 2020, Elsevier.

Realizing the repeatable ability of self-healing materials could ensure response reliability and mechanical stability of the sensors of human–machine interactions. Cao et al. describe a kind of hydrogen bonding sensor that is highly sensitive and capable of repeatable self-healing [55]. The sensor is combined with signal processing software and attached to special places that can detect not only subtle human motions but also wrist motion. In addition, the sensor can be directly attached to the throat to recognize different words due to their characteristic signal curves (Figure 2a). The high sensitivity of the sensor, which enables it to display the data by 15 s at the most, is achieved by C-CNC, constructing a brittle but effective nanostructure 3D conductive network [103]. Meanwhile, C-CNC can build a supramolecular multiple-hydrogen-bonding network to achieve repeated self-healing abilities. The self-healing ability of the material would not be influenced even after bending over 2000 cycles. The sensor can still provide highly sensitive and reliable results under a high-intensity working environment for the system of human–machine interaction. The cold resistance of self-healing material could be achieved by a special ionic-crosslinking phase. Miao et al. designed a “C-I hydrogel” that is conductive, cold-resistant and elastic and can be used as a potential bionic skin for human–machine interaction control [104]. The sensor can be attached to the human skin as a soft sensor. The resistance of the sensor would change with the difference of the finger’s bending, which could be transferred to corresponding digital signals and guide the motion of the electric steering engine by encoding these digital signals (Figure 2b).

The ionic-crosslinking phase generated by the presence of K^+^ and locust bean gum gives the hydrogel cold-resistance properties [105]. When the sensor attaches to the human finger skin, the self-healing property makes it not fall off even under the repeating bending motion. The notch insensitivity suggests that the gap would not extend after unloading. Furthermore, the cold resistance of the C-I hydrogel was able to keep its softness and stretchability at −10 °C, which can help realize the work of a human–machine interaction system in a low-temperature environment. Another enabling factor of the self-healing e-skin is biocompatibility. This design ensures the e-skin can be contacted to the human skin directly without adverse reactions. Zhao et al. fabricated a poly(N-vinylpyrrolidone)/gallic acid (PVP/GA) composited hydrogel, which exhibits nice self-adhesive, self-healing and biocompatible properties [106]. The hydrogels adhering to human skin could detect physiological activity signals according to the change in resistance signals (Figure 2c). The high cell affinity of the PVP/GA hydrogels is ascribed to the presence of the biocompatible pyrogallol groups, which are known for absorption on the hydrogel surface, leading to a better environment for cell attachment and proliferation [107]. The biocompatibility of the sensor could allow it to be attached to human skin directly and detect human activities in real-time so that the biocompatibility sensor could provide reliable and sensitive data to the HMI systems.

### 3.3. Soft Robots

Soft robots are new types of robots that have been developed in recent years [108,109]. Compared to traditional hard robots, soft robots made of soft organic elastomers require fewer parts for the system and can produce surprisingly complex movements based on their ability to deform under low stress [110,111]. Since the constructions of soft robots are almost entirely made of flexible materials, they are suitable for some complex, uncertain scenarios, such as cuts and perforations in uncontrolled and unpredictable shapes caused by sharp objects [112]. Complex scenarios demand soft robots to be able to heal themselves after sustaining damage. Currently, the main polymers used in soft robotics are thermo-reversible polymers, which can repeatedly heal macroscopic damage in elastomers with relatively high mechanical strength and high healing efficiency. Since photo-reversible polymers can heal only damage of a limited depth (<0.2 mm), they are not currently used in soft robots [55]. In addition, for many application scenarios, the general requirement of self-healing time is in hours, but for soft robots alone, the parameter of self-healing time is very important (generally in the level of seconds).

**Figure 3:**
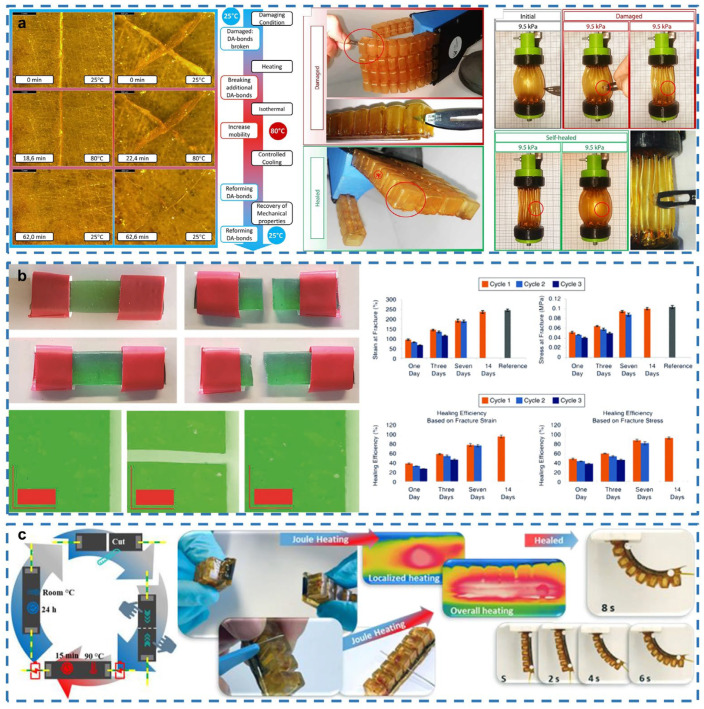
Self-healing soft robots. (**a**) Schematic of the self-healing cycle of DA polymers and validation of the self-healing ability in practice. Reprinted with permission from [112]. Copyright 2017, American Association for the Advancement of Science. (**b**) Testing autonomous healing at room temperature and the self-healing efficiencies. Reprinted with permission from [114]. Copyright 2020, IEEE. (**c**) A healable resistive-heater self-healing soft robot. Reprinted with permission from [115]. Copyright 2022, IEEE.

Due to the complexity of soft robotics application scenarios, soft robots can be easily cut and perforated by sharp objects [113]. Terryn et al. demonstrate that soft robots are capable of self-healing using DA polymers with thermo-reversible covalent networks for the development of soft grippers, soft hands and artificial muscles in self-healing soft pneumatic actuators (Figure 3a) [112].

Devices fabricated with DA thermally reversible elastomers have been used on millimeter scalpels, requiring heating of the parts to 80 °C after 40 molecules, followed by placement at 25 °C at room temperature, with full recovery of performance after 24 h. Although full recovery of performance can be achieved, the need to heat the temperature to 80 °C limits their application. Terryn et al. propose a new network based on DA polymers [114]. A low maleimide-to-furan ratio is used in the fabrication of DA polymers, which reduces the crosslink density and improves the mobility of the molecules and can enable DA-fabricated devices to accomplish self-healing (full recovery of properties) at room temperature (Figure 3b). More importantly, the recovery time depends on the location of the damage, ranging from seconds to days. Although good self-healing can be achieved without heating, defects and longer recovery times may be present. For thermally reversible self-healing networks, higher temperatures can accelerate the healing process. Tabrizian et al. developed a self-healing soft actuator integrated with a self-healing electronic heater in which the actuator consists of a DA thermally reversible elastomer with a healing capability [115]. DA filled with 20% carbon black serves as a substrate of the heater, providing conductive properties for Joule resistance heating. The heater will behave as a localized heating source in severely damaged areas, and it takes only 15 min at 35 v to achieve more than 96% healing efficiency (Figure 3c). This strategy makes the self-healing material promising for a wide range of applications in soft robotics.

### 3.4. Other Applications

**Figure 4:**
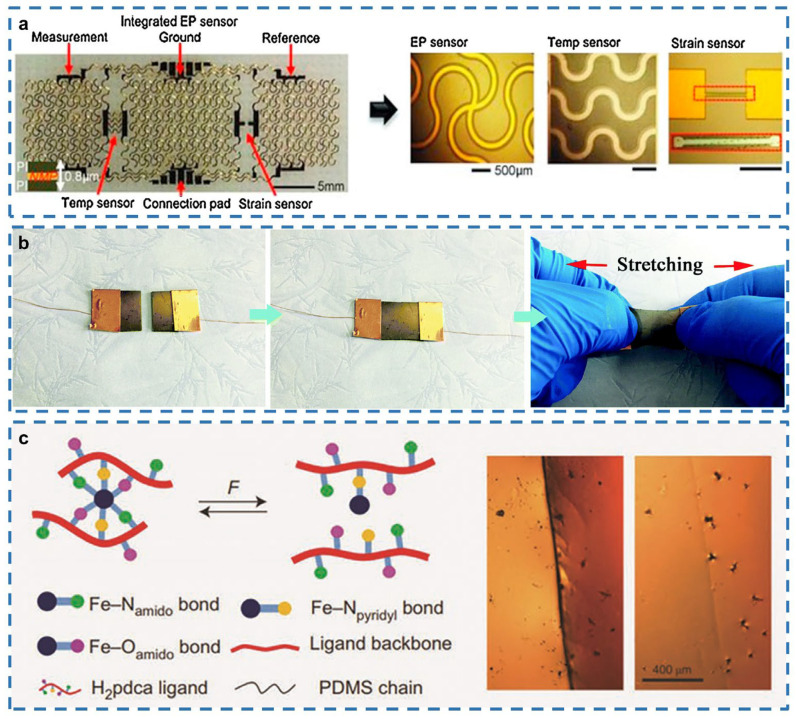
Self-healing e-skin devices. (**a**) A multifunctional epidermal electronic system. Reprinted with permission from [116]. Copyright 2017, Elsevier. (**b**) Stretchable and self-healable electrical sensors for surface texture discernment and biosignal monitoring. Reprinted with permission from [117]. Copyright 2019, WILEY-VCH. (**c**) Autonomous self-healing elastomer using metal coordination. Reprinted with permission from [118]. Copyright 2019, The Royal Society of Chemistry.

Regarding self-healing e-skin progress, more application scenarios are being explored. For human skin, sensing temperature is an important aspect of tactile sensing. In the field of thermal sensors, commercial temperature sensors mainly utilize the thermal resistance effect of pure metal or ceramic-based semiconductors [116], the resistivity of which varies with temperature due to changes in mobility and/or carrier density (Figure 4a). However, they are not compatible with e-skin devices due to their inherent rigidity. Most e-skin devices use polymers as substrates, and the properties of polymers depend to some extent on temperature due to their mobility that is easily affected by temperature. Among them, conductive polymers have high sensitivity due to their temperature-dependent conductivity and thus can be used as sensing materials for sensors. In temperature monitoring, e-skin is prone to damage in a complex external environment; thus, self-healing is valued as an important property in thermal sensors. Rapid self-healing allows e-skin to maintain high sensitivity and durability during thermal monitoring.

The e-skin has good sensing performance with ultra-fast response time, and it can monitor smooth pulses at high frequencies to achieve real-time determination of the pulse response of the wrist artery [117]. In addition, human sweat can provide a lot of human health information, and diagnoses based on sweat can be an effective non-invasive monitoring method to gather information for understanding the health condition of the human body (Figure 4b). E-skin can capture, store and analyze sweat by fabricating skin-mountable sweat sensors and quantitatively measuring sweat rate, total sweat volume, pH, chloride and lactate concentrations. The gathered information can be sent wirelessly via near-field communication technology [118]. A good self-healing ability enables an electronic device to complete repairing itself and recovering its performance, ensuring its stable operation in a short time after being injured by external effects such as twisting, squeezing and stretching (Figure 4c). This makes self-healing e-skin promising for medical monitoring applications.

## 4. Summary

The common mechanisms for self-healing materials are extrinsic self-healing and intrinsic self-healing. Extrinsic self-healing mechanisms usually require additional healing agents to help repair the damaged part. Their practical application, consequently, is limited by the fact that healing agents are easily depleted, multiple healings of the same site are difficult and larger damages need to be repaired by encapsulating the healing agent in hollow fibers or vascular systems. Compared with the external self-healing mechanism, the internal self-healing mechanism will be the mainstream direction of future self-healing technology, which promotes a self-healing ability that is more stable and reliable and can achieve rapid multiple reversible healings through intrinsically reversible dynamic covalent bonding or dynamic non-covalent bonding. It is worth noting that in some materials, the conditions for non-covalent and covalent bonding self-healing are similar and not always limited to reversible conversion of individual chemists. In addition, different modifications of self-healing polymeric materials can lead to unique properties such as stretchability, meeting other properties such as mechanical properties, self-healing rate and biocompatibility required for various applications.

Although considerable progress has been made in self-healing materials, research on electronic devices with self-healing capabilities is still in its infancy. Most current self-healing e-skins are able to achieve almost complete self-healing but with a short life cycle, which can lead to significant waste generation. In addition, designing conductive ionic skins with good elasticity, complete self-healing and strain enhancement is difficult due to stress relaxation and strain hardening. Excitingly, e-waste has now attracted the attention of scholars and there has been some progress in that direction, which can make it possible to recycle some of the raw materials of electronic devices. Moreover, some scholars have designed conductive ionic skins with good self-healing and elasticity by introducing entropy-driven supramolecular amphiphilic ion-formed e-skins in addition to the development of materials with excellent self-healing capabilities. The future of e-skin will still present more challenges.

## Data Availability

Non applicable.
